# Comparative Studies of the Confined Effect of Shear Masonry Walls Made of Autoclaved Aerated Concrete Masonry Units

**DOI:** 10.3390/ma16175885

**Published:** 2023-08-28

**Authors:** Radosław Jasiński, Tomasz Gąsiorowski

**Affiliations:** 1 Department of Building Structures and Laboratory of Civil Engineering Faculty, Faculty of Civil Engineering, Silesian University of Technology, Akademicka 5, 44-100 Gliwice, Poland; radoslaw.jasinski@polsl.pl; 2 Department of Building Structures, Faculty of Civil Engineering, Silesian University of Technology, Akademicka 5, 44-100 Gliwice, Poland; 3 Expertise Office TMG Sp. z o.o., Św. Stanisława 14/Ip, 32-540 Trzebinia, Poland

**Keywords:** masonry structures, shear walls, autoclaved aerated concrete AAC, confined masonry

## Abstract

Confined walls are popular in areas exposed to seismic action. The advantage of such structures is increased load-bearing capacity, ductility, and energy dissipation. Confined masonry walls are also used to restrain the intensity of cracking and improve load-bearing capacity in areas exposed to seismic action. This paper describes the research on 18 confined walls and presents a comparison with research on unconfined walls (referenced models). The confined models were classified into three series: HOS-C-AAC—without openings and with confining elements around the perimeter; HAS-C1-AAC with a centrally positioned opening and circumferential confinement; and HAS-C2-AAC with a centrally positioned window opening and additional confinement along the vertical edges of the opening. The area of the window opening was 1.5 m^2^. All walls were made of autoclaved aerated concrete (AAC) masonry units of the nominal density class of 600. The walls were tested under initial compressive stresses *σ*_c_ = 0.1; 0.75; and 1.0 N/mm^2^. The reference models without confinement (six models of the series HOS-AAC without openings and the series HAS-AAC with openings) were prepared from the same masonry units, had almost the same outer dimensions, and were tested under the same initial compressive stresses *σ*_c_. The analysis was performed for the morphology of cracks, stress values at the moment of cracking and failure, stiffness, and angles of shear strain. The morphology of cracks was found to depend on initial compressive stresses and the presence of an opening. A significant increase in compressive stress leading to cracks and failure stresses was observed with increasing values of initial compressive stresses. As the wall behavior was clearly non-linear, the bilinear relationship described by energy dissipation *E*, stiffness at the moment of cracking *K*_cr_, and maximum displacement *u*_u_ was proposed to be included in the engineering description of the relationship between horizontal load and displacement of confined walls. Confinement along the vertical edges of the opening having an area of 1.5 m^2^ (acc. to EN 1996-1-1) increased the maximum forces *P*_max_ by ca. 45% and marginally affected the ductility of the wall when compared to the elements with circumferential confinement.

## 1. Introduction

The low compressive and tensile strength of the wall made of AAC masonry units results in the relatively early formation of superficial cracks, and its load-bearing capacity is considerably lower than that of other walls made of (concrete, silicate, ceramic) masonry units with greater load-bearing capacity. Therefore, there is a continuous search for methods of improving cracking and failure stresses, reducing the width of cracks, and eliminating brittle failure (increasing ductility). This aspect is particularly relevant to stiffening walls exposed to vertical and shear loading and to stiffening walls with window or door openings [1,2]. This stress level in unreinforced walls [3,4,5] results in the early formation of superficial cracks, loss of stiffness, and consequently excessive displacements, which deteriorate limit states and increase eccentricity values in walls with mainly vertical loading. As in the case of reinforced concrete structures, steel reinforcement [6,7] or reinforcement made of different types of fibers [8,9] are used to eliminate excessive cracks or damage. Crack resistance in walls with openings can be increased by reinforcement [10,11,12,13,14], in vertical cores, or by confining with reinforced concrete elements [15,16]. Due to the proper sequence of its construction [17,18,19,20], such a structure ensures the full interface between masonry and reinforced concrete. Considering the seismic actions, the effectiveness of confining the masonry wall made of autoclaved aerated concrete units has been confirmed by numerous research and theoretical analyses [15,16,21,22,23,24,25]. However, there are no papers presenting relevant tests on the behavior of confined walls under monotonic loading [26], where confinement is applied only to improve load-bearing capacity and deformability. The load-bearing capacity of confined walls is affected by many factors, which include the properties of masonry components (mortar and masonry units), proportions of dimensions, values of initial compressive stress, and the static diagram of walls. The impact of confinement on the unconfined walls cannot be clearly indicated, but test results presented in the overview study [27] show that this impact is positive. According to some tests [28,29,30,31], vertical confining elements can increase the load-bearing capacity of the confined wall even by 40%. However, the impact of confinement on walls with openings has been barely recognized. There have been a few tests in this area, and they are the papers [32,33,34,35,36], in which observations were made not only on known factors but also on vertical confining elements and the shape and size of openings. The valid European provisions EN 1996-1-1 [37] contain crucial information on constructing confined walls regarding, among other things, the arrangement of reinforced concrete elements and the reinforcement ratio. The clause on the required confinement of all openings with an area greater than 1.5 m^2^ complicates the common application.

This paper is a kind of detailed report and presents results from testing confined walls with and without openings, whose main aim was to demonstrate the effect of confinement on fundamental mechanical parameters of the masonry. Confined walls with openings had simpler confining elements than recommended by the standard [37], positioned only along the vertical edges of openings. The paper also describes the analysis of cracking morphology, the destruction mechanism, shear deformation, and stiffness of walls. The bilinear model describing the load-displacement relationship was proposed for engineering solutions. The factors that have a significant impact on these parameters include the geometry of walls and confining elements and the parameters of masonry components [38]. Contrary to unreinforced and unconfined walls, the yield strength of such structures dissipates energy and suppresses vibrations. For structures under monotonic load, yield strength is particularly important and useful to the design process, which can employ the truss methods [39,40,41]. In relation to previous tests on confined walls [25] conducted by the author, this paper presents more detailed test results, describes tests on walls with and without openings and with different types of confinement, and also proposes the bilinear model of wall behavior.

## 2. Research Models

Tests were performed on 18 confined walls made of autoclaved aerated concrete (AAC) units with a density of 600 (*f*_b_ = 3.65 N/mm^2^) using ready-mixed mortar for thin joints (*f*_m_ = 6.1 N/mm^2^) with unfilled head joints. The compressive strength of masonry determined in accordance with EN 1052-1:2000 [42] was *f*_cm_ = 2.97 N/mm^2^, and the modulus of elasticity was *E*_cm_ = 2041 N/mm^2^. Shear strength determined in accordance with EN 1052-3:2004 [43] was *f*_v0_ = 0.31 N/mm^2^, and shear modulus for walls under diagonal compression determined in accordance with the standard ASTM E519-81 [44] was *G* = 475 N/mm^2^.

**Figure 1:**
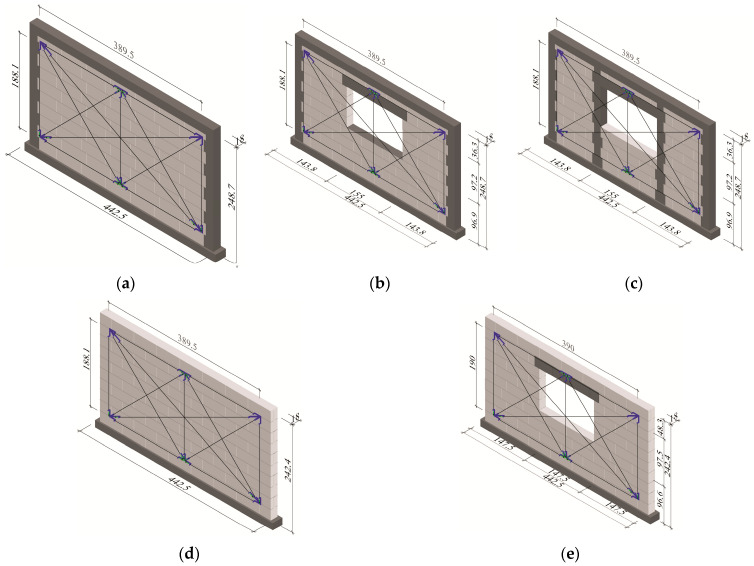
Geometry of research models: (**a**) confined walls of the series HOS-C-AAC without openings [26], (**b**) confined walls of the series HAS-C1-AAC with openings, (**c**) confined walls of the series HAS-C2-AAC with openings, (**d**) unconfined (reference) walls of the series HOS-AAC without openings [26], (**e**) unconfined (reference) walls of the series HAS-AAC with openings (dimensions are given in centimeters).

The models were divided into three series and marked as HOS-C-AAC (Figure 1a), HAS-C1-AAC (Figure 1b), and HAS-C2-AAC (Figure 1c). They were prepared as enclosed models. All walls had identical external dimensions: *l* = 4.43 m, *h* = 2.49 m, and thickness *t* = 180 mm. The walls were tested under initial compressive stresses *σ*_c_ = 0.1; 0.75, and 1.0 N/mm^2^ (0.025–0.25)*f*_b_. The unreinforced models [45] made of the same materials with a length *l* = 4.43 m, a height *h* = 2.43 m, and an identical thickness *t* = 0.18 m were tested and analyzed to compare the effect of confinement. The unreinforced walls were divided into two series. Four models of the series HOS-AAC (Figure 1d) were prepared and tested under compressive stresses *σ*_c_ = 0.1, 0.75, and 1.0 N/mm^2^ (0.025–0.25)*f*_b_, and two models of the series HAS-AAC (Figure 1e) were tested under stresses *σ*_c_ = 0.1 and 1.0 N/mm^2^ (0.025–0.25)*f*_b_. All models were made with thin joints and unfilled head joints.

Reinforced concrete confinement in the confined models of the series HOS-C-AAC was prepared as two vertical cores running along the wall edge and along the top and bottom horizontal members. Reinforced concrete horizontal members were connected with toothings in a mesh having a minimal overlapping of 50 mm. Because of the adjustment of toothings, the width of the cross-section of cores was changing within the range from 180 mm to 230 mm (*A* = 0.041–0.032 m^2^), whereas their thickness was the same and equal to 180 mm.

**Figure 2:**
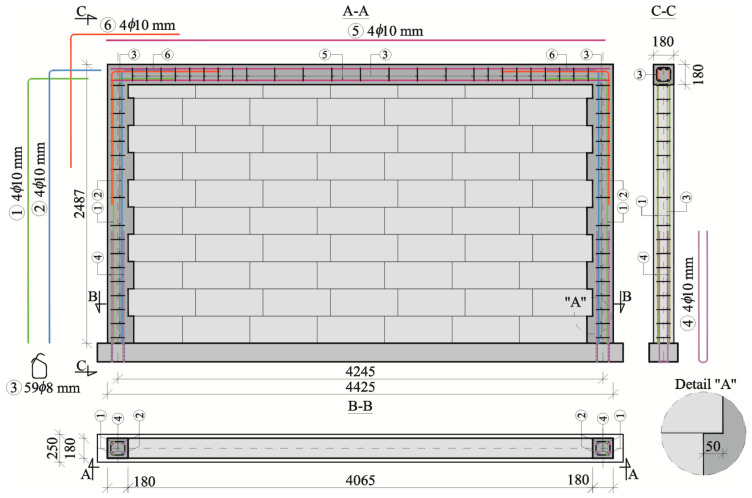
Structure of confined walls of the series HOS-C-AAC without openings acc. [26] (dimensions are given in millimeters).

Confined elements of the research models were performed in accordance with guidelines specified in the standard EN 1996-1-1 [37], providing the minimum ratio of longitudinal reinforcement ρ_min_ = 0.8% and reinforcement cover *c*_nom_ = 25 mm. The confining elements in the model of the series HOS-C-AAC, which are shown in Figure 2, were made of steel grade B500SP.

Vertical cores were reinforced with bars with a diameter of 10 mm (rebars No. 1 and 2) placed in each corner, which provided the overall ratio of reinforcement ρ = 1.29% > ρ_min_ = 0.8%. The stirrups made of bars with a diameter of 8 mm (rebar No. 3) were placed along the core length every 250 mm and every 125 mm in the sections of reinforcement overlapping. Th bottom parts of the rebars No. 1 and 2 lapped with the “starter” rebars No. 4, having a diameter of 10 mm, over a distance of 1000 mm.

The bottom horizontal grit prepared as a precast unit (with a cross-section *b* × *h* = 250 × 165 mm and a length of 4600 mm) represented the ring beam or the floor reinforced with rebars with a diameter of 16 mm placed in each corner. The stirrups, having a diameter of 10 mm, were arranged at a regular spacing of 150 mm along the whole length of the beam.

The upper horizontal grit, having dimensions of 180 × 180 mm, was reinforced with straight rebar No. 5 placed in each corner. Like in the vertical cores, stirrups No. 3 with a diameter of 8 mm and a spacing of 250 mm, concentrated to 125 mm, were arranged transversely in places of connecting the reinforcement with vertical cores. The upper beam and vertical cores were connected with rebar No. 6 (ø10) hooked at a right angle. The laps along vertical cores were 1250 mm long, and those along horizontal confining elements were 580 mm long. Confining elements were made of ordinary concrete with a compressive strength of *f*_c,cube_ = 25.1 N/mm^2^. The reinforced concrete confining elements were performed by shaping the vertical edges of the masonry into a sawtooth shape—cf. detailed drawing “A” in Figure 2. The depth of each bond was 50 mm = ~0.21 *h*_u_, which was smaller than the value of 100 mm recommended in the standard EN 1996-1-1 [37].

**Figure 3:**
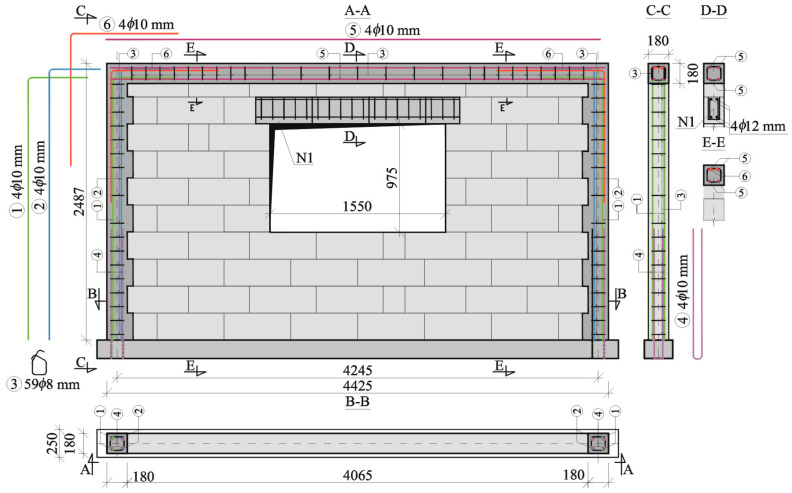
Structure of confined walls of the series HAS-C1-AAC with window openings (dimensions are given in millimeters).

The models of the series HAS-C1-AAC had a centrally positioned rectangular opening with a height of 0.972 m, a length of 1.55 m, and a surface area of 1.50 m^2^. Confinement was along the whole perimeter, as in the models of the series HOS-C-AAC. Vertical reinforcement of cores and horizontal members was characterized by the same geometry and shape as in the walls of the series HOS-C-AAC. Confining reinforced concrete elements were made of concrete with a compressive strength of *f*_c,cube_ = 25.2 N/mm^2^. Reinforced concrete precast lintels made of AAC profiles were used above the window opening. The test elements of the series HAS-C1-AAC are shown in Figure 3.

**Figure 4:**
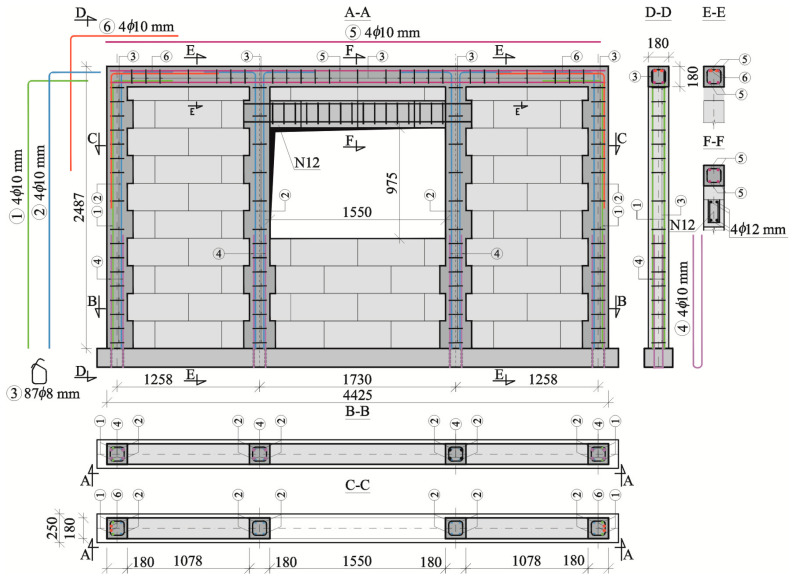
Structure of confined walls of the series HAS-C2-AAC with window openings (dimensions are given in millimeters).

The models of the series HAS-C2-AAC had a centrally positioned opening with the same geometry as the models of the series C1. Confining elements were arranged along the wall perimeter and along the vertical edges of the window opening. The geometry and shape of the reinforcement of external cores were the same as in the models of the series HOS-C-AAC and HAS-C1-AAC—cf. Figure 2 and Figure 3. Confining reinforced concrete elements were made of concrete with a compressive strength of *f*_c,cube_ = 24.8 N/mm^2^. Internal cores at the vertical edge of the opening were reinforced with four rebars with a diameter of 10 mm (rebar No. 2 in Figure 4) running in each corner of the section. The rebars were anchored at the top edge of the horizontal member over a length of 600 mm. Stirrups (rebar No. 3 in Figure 4) were made of rebars with a diameter of 8 mm, made of steel B500SP, spaced at 250 mm in the central section of a core and at a spacing of 125 mm in the section of overlapping. “Starter” rebars No. 4 (ø10) shown in Figure 4, lapped with rebars No. 2 over a distance of 1000 mm, were used to connect internal cores with horizontal members. The bottom horizontal member, on which the masonry was erected and reinforcement of vertical cores was anchored, was prepared in the same way as for other series, that is, in the form of a precast beam with a rectangular section *b* × *h* = 250 × 165 mm and a length of 4600 mm. The cross-section of the upper ring beam had a square shape with a side of 180 mm and was reinforced with steel rebars No. 5 (Figure 4) with a diameter of 10 mm arranged symmetrically in the cross-section. The wall had stirrups—rebars No. 3 (Figure 4)—with a diameter of 8 mm at a spacing of 125 mm in the sections that overlapped and of 250 mm in the central sections. The upper ring beams and vertical cores were connected with rebars No. 6 (Figure 4) hooked at an angle of 90°. The length of laps in the upper beams was 1250 mm and 580 mm in the vertical cores.

Partially precast, reinforced concrete lintels with commercial “U” type lintel blocks, which were used as stay-in-place formwork of reinforced concrete core, were placed above the window openings in the models of the series HAS-AAC, HAS-C1-AAC, and HAS-C2-AAC. Lintel blocks made of aerated concrete had a length of 500 mm and a width of 180 mm. The thickness of the two walls of the webs and the bottom flange was equal to 40 mm. The core was filled with concrete after placing lintel blocks in the target position. Lintels had a rectangular section with dimensions *b × h* = 180 × 240 mm and different lengths determined by the type of opening. A reinforced concrete core, in which reinforcement was placed, had a width *b* = 100 mm and a height *h* = 160 mm. The lintel N1, which was in the models of the series HAS-C1-AAC (cf. Figure 3), had a length of 1800 mm, including completely covered longitudinal reinforcement. The concrete core was reinforced with precast steel meshes composed of two longitudinal rebars having a diameter of 12 mm and made of steel B500SP. The compressive strength of the applied concrete infill was *f*_c,cube_ = 27.1 N/mm^2^. Transverse shear reinforcement applied to each mesh consisted of rebars with a diameter of 8 mm at a spacing of 100 mm perpendicularly joined by welding to the longitudinal bars of the mesh. The lintels N12 used in the models of the series HAS-C2-AAC (cf. Figure 4) were 1550 mm long, with rebars of the longitudinal reinforcement terminated outside on both sides, which were used to obtain a solid form with confining mandrels near openings. The compressive strength of the applied concrete infill was *f*_c,cube_ = 24.1 N/mm^2^. The reinforcement of the longitudinal and transverse lintel N1 had the same structure as the lintel N12.

The process of preparing all confined models was described in detail in the paper [26] and took place in four stages:

- Stage I—placing starter rebars in the bottom horizontal member, filling openings with cement mortar,
- Stage II—building masonry on the bottom horizontal member, keeping lapped toothings, placing reinforcement of vertical cores on starter rebars,
- Stage III—shuttering and concreting vertical cores to a height of ca. 1.5 m. Then, stirrups were added to the upper parts of the cores without concrete, and later, the top horizontal members were reinforced. Continuity of reinforcement in the wall corners was achieved using bars bent at the right angle.
- Stage IV—shuttering and concreting of the top parts of cores and horizontal members. Elements were stripped after 28 days and prepared for testing.

## 3. Testing Technique

**Figure 5:**
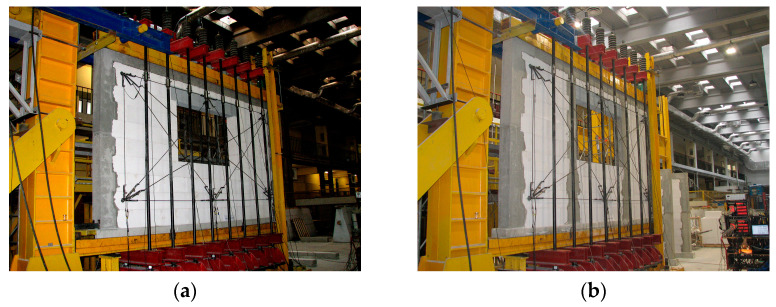
Overall view of the test stand: (**a**) model of the series HAS-C1-AAC; (**b**) confined model of the series HAS-C2-AAC.

The walls were tested at the author’s original test stand, which can be used to perform simultaneous tests on shearing and compression of full-size walls [26,46]. The test stand can be used to test walls in a fixed static scheme, which means that the bending moment changes the behavior in contrast with shear walls in the cantilever scheme. The horizontal force with a maximum value of 3000 kN was produced by a hydraulic actuator, and initial compressive stresses were generated by the system of eight tendons equipped with hydraulic actuators. A view of the test stand and confined elements with window openings is illustrated in Figure 5.

**Figure 6:**
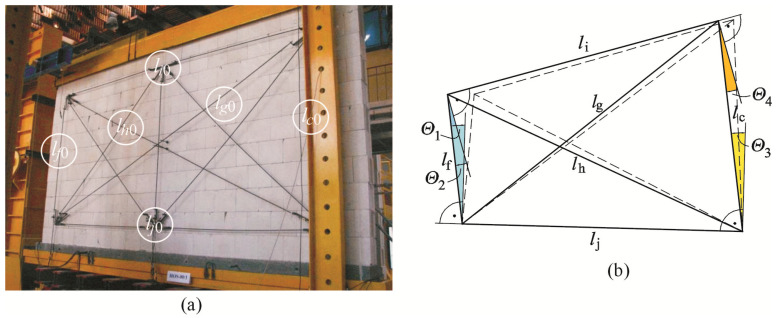
The frame structure for measuring shear angles: (**a**) symbols of measuring bases [26], (**b**) shear angles *Θ_j_* (*Θ*_1_, *Θ*_2_, *Θ*_3_, *Θ*_4_).

Shear strains of walls were determined on the basis of changes in the section length *l*_c0_, *l*_f0_, *l*_i0_, *l*_j0_, *l*_g0_, *l*_h0_—Figure 6, placed on both sides of the analyzed model. Changes in the length of reference frames were recorded with LVDT (PELTRON S.A.) PJX-20 with an accuracy of ±0.002 mm and a range of indications of 20 mm.

As in the previous paper [26], shear deformations of the wall were determined in the elastic phase (prior to cracking) and after the formation of the cracks. Global angle of shear strain was a term used in the elastic phase, whereas global angle of shear deformation was used in the post-cracking phase (until failure). The mean value of the angle of shear deformation of the wall *Θ_i_*, (at the *i*-th level of loading) was determined from the following equation:

(1) $$\Theta_{i}=\frac{1}{n}\left(\sum_{j=1}^{n=4}\left|\Theta_{j}\right|\right).$$

where *Θ_j_* is the angle of shear strain (Figure 6b) *Θ*_1_ from a triangle composed of the following sections: *l*_f_, *l*_h_, *l*_j_, *Θ*_2_ from a triangle composed of the following sections: *l*_f_, *l*_i_, *l*_g_, *Θ*_3_ from a triangle composed of the following sections: *l*_j_, *l*_c_, *l*_g_, *Θ*_4_ from a triangle composed of the following sections: *l*_c_, *l*_i_, *l*_h_.

Shear stresses *τ*_i_ were calculated from the following equation:

(2) $$\tau_{v,i}=\frac{H_{i}}{A_{h}}.$$

where *H_i_* is the horizontal shearing force and *A_h_* = 4.43 × 0.18 = 0.797 m^2^—area of the wall cross-section.

The general stiffness of a wall, *K_i_* (at the *i*-th level of loading), was calculated from the following relationship:

(3) $$K_{i}=\frac{H_{i}}{u_{i}}=\frac{\tau_{i}}{\Theta_{i}}\frac{A_{h}}{h}.$$

The load recorded at the time of formation of cracks having a width of *w* = ~0.1 mm was defined as the cracking force *H*_cr_, and the corresponding shear strain was defined as the cracking stresses *τ*_cr_, and the angle at the time of cracking *Θ*_cr_. The greatest recorded force was defined as the ultimate force *H*_u_, and the corresponding stress and deformation were defined as the failure stresses *τ*_u_ and the angle of shear deformation *Θ*_u_. Horizontal displacements were determined according to the following relationship:

(4) $$u_{i}=\Theta_{i}h.$$

Dissipated energy *E*_obs_ was calculated as the area below the load-displacement curve according to the following relationship:

(5) $$E_{\mathrm{obs}}=\int_{0}^{u}Hudu=\sum_{i=1}^{b}\frac{1}{2}\left(H_{i+1}-H_{i}\right)\left(u_{i+1}-u_{i}\right)=\frac{1}{2}A_{h}h\sum_{i=1}^{b}\left(\tau_{v,i+1}-\tau_{v,i}\right)\left(\Theta_{i+1}-\Theta_{i}\right).$$

## 4. Test Results

### 4.1. Mechanism of Cracking and Failure of the Models without Openings

**Figure 7:**
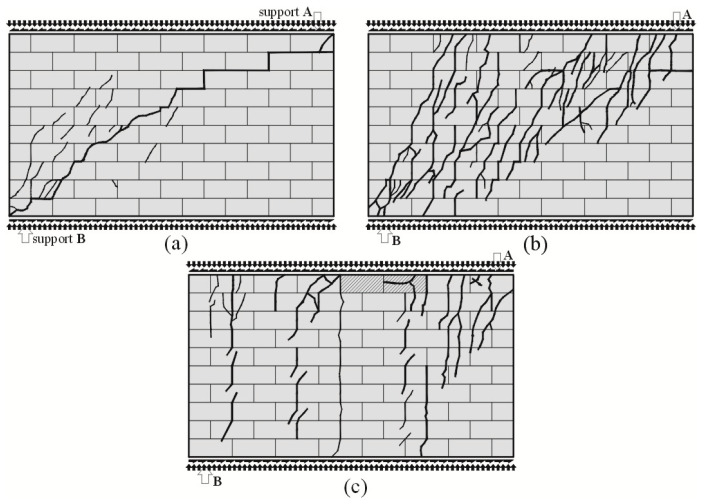
Cracking patterns for walls of the series HOS-AAC at the time of failure acc. to [26]: (**a**) wall under initial compressive stress up to *σ*_c_ *=* 0.1 N/mm^2^, (**b**) wall under initial compressive stress up to *σ*_c_ *=* 0.75 N/mm^2^, (**c**) wall under initial compressive stress up to *σ*_c_ = 1.0 N/mm^2^.

Superficial cracks in unreinforced stocky walls (the longest ones) of the series HOS-AAC developed in the central part of the wall [26]. In the wall subjected to minimal compressive stress, a single diagonal crack was running through the bed and head joints at the interface between the masonry units (Figure 7a). Many diagonal cracks were formed in the masonry units in the bottom part of the wall above support B. Also, in the wall, under a compressive load of 0.75 N/mm^2^, the first cracks in head and bed joints were found in the central part of the wall—Figure 7b. At increasing shear loads, diagonal cracks propagated towards the bottom and top edges of the wall, as did many vertical cracks. In the walls under maximum compressive stress up to 1.0 N/mm^2^ (Figure 7c), an increase in shear load resulted in a series of vertical cracks slightly deflected at the support A. Masonry crushing at the top edge was found locally.

**Figure 8:**
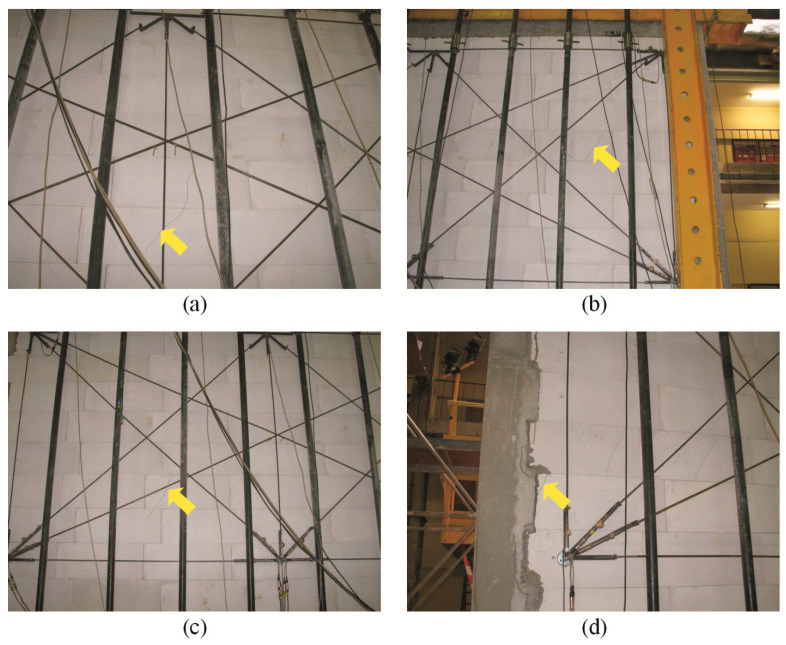
Superficial cracks in confined walls of the series HOS-C-AAC under initial compressive stress up to *σ*_c_ = 0.1 N/mm^2^ [26]: (**a**) first cracks in the bottom corner of the wall; (**b**) first cracks in the top corner of the wall; (**c**) first cracks in the central area of the wall; (**d**) cracks at the interface between the masonry and confining reinforced concrete elements.

**Figure 9:**
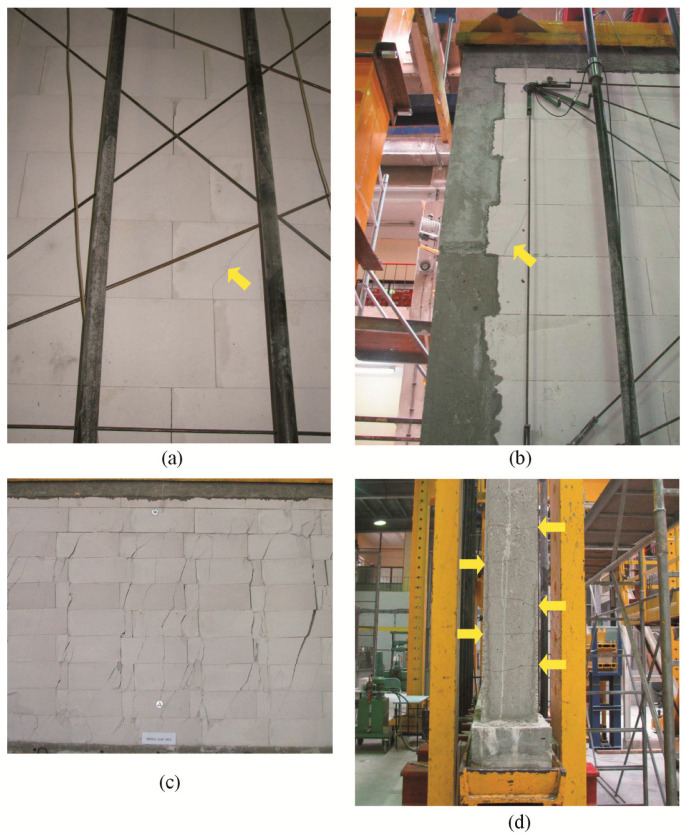
Cracks in confined walls of the series HOS-C-AAC under initial compressive stress up to *σ*_c_ = 0.75 N/mm^2^ and *σ*_c_ = 1.0 N/mm^2^: (**a**) first cracks in the central area of the wall; (**b**) first cracks at the interface between the masonry and confining reinforced concrete elements; (**c**) vertical cracks in the central part of the wall; (**d**) horizontal cracks in confining reinforced concrete elements.

For confined walls subjected to shearing under an initial compressive stress equal to 0.1 N/mm^2^, the formation of cracks was noticed in the area of the corner diagonals of the walls (Figure 8a,b). As the load increased, superficial cracks propagated towards the central areas of the walls. In central areas of the walls, there were inclined and vertical cracks near head joints in masonry units (Figure 8c). Apart from the above, the connection between the masonry and vertical confining elements was damaged (Figure 8d). In the walls under initial compressive stress up to 0.75 N/mm^2^, first cracks were observed in central areas of the walls (Figure 9a). An increase in load caused a crack in the connections between the masonry and vertical cores (Figure 9b). When shearing was accompanied by an initial compressive stress equal to 1.0 N/mm^2^, the first visible cracks were found in the central areas of the wall, however they were nearly vertical and not inclined. An increase in load caused cracks at the interface between the masonry and confining elements and caused the development of previous vertical cracks in the masonry (Figure 9c). Also, bending resulted in horizontal cracks in vertical cores (Figure 9d).

**Figure 10:**
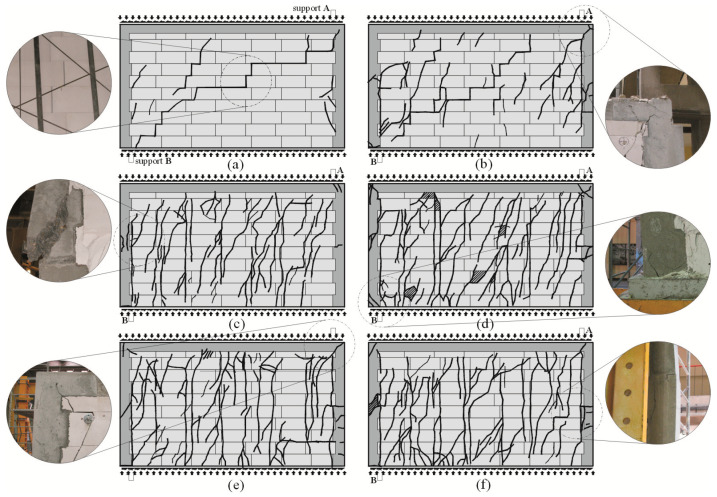
Images of cracks in confined walls of the series HOS-C-AAC at failure acc. to [26]: (**a**) the wall HOS-C-AAC-010/1 under initial compressive stress up to *σ*_c_ = 0.1 N/mm^2^, (**b**) the wall HOS-C-AAC-010/2 under initial compressive stress up to *σ*_c_ = 0.1 N/mm^2^, (**c**) the wall HOS-C-AAC-075/1 under initial compressive stress up to *σ*_c_ = 0.75 N/mm^2^, (**d**) the wall HOS-C-AAC-075/2 under initial compressive stress up to *σ*_c_ = 0.75 N/mm^2^, (**e**) the wall HOS-C-AAC-10/1 under initial compressive stress up to *σ*_c_ = 1.0 N/mm^2^, (**f**) the wall HOS-C-AAC-10/2 under initial compressive stress up to *σ*_c_ = 1.0 N/mm^2^.

Regardless of the initial compressive stress values, the failure mechanics were not rapid. Inclined cracks in the walls under minimum compressive stress (*σ*_c_ = 0.1 N/mm^2^) covered nearly the whole length of the wall diagonal (Figure 10a,b). Moreover, damage was found in the upper corners of the walls, and horizontal cracks were observed in construction joints (Figure 10b).

In the case of other walls compressed to *σ*_c_ = 0.75 N/mm^2^ and 1.0 N/mm^2^, considerably fewer cracks were formed at the time of failure, apart from previous vertical cracks, which considerably deflected from their vertical direction. Inclined/horizontal shear failure was noticed in horizontal construction joints in vertical confining elements (Figure 10c). Apart from the above, inclined cracks were found in the connections between horizontal and vertical confining elements (Figure 10d). Vertical confining elements were found to undergo elastic deformation, and cracks developed at the construction interface at the mid-height of the wall (Figure 10e,f).

### 4.2. Mechanism of Cracking and Failure of Models with Openings

**Figure 11:**
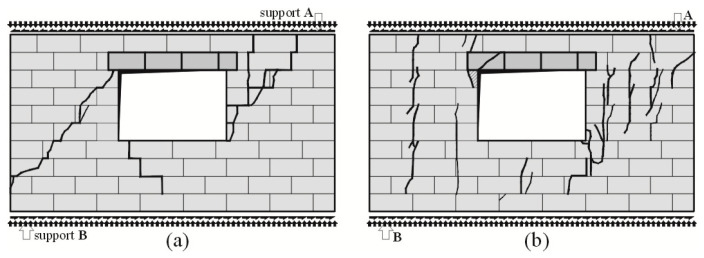
Cracking patterns of unconfined walls of the series HAS-AAC with an opening at failure: (**a**) wall under initial compressive stress up to *σ*_c_ = 0.1 N/mm^2^, (**b**) wall under initial compressive stress up to *σ*_c_ = 1.0 N/mm^2^.

First cracks in the reference wall with an opening, subjected to compression up to 0.1 N/mm^2^, were observed in the tension corner of the window opening above the support B and then in the central area of the pillar (Figure 11a). A similar mechanism of cracking was observed in the model compressed up to 1.0 N/mm^2^—the first cracks were noticed above the support B and then in the window pillar. It should be emphasized that the cracks in the pillar were almost vertical (Figure 11b).

An increase in horizontal loading caused the development of primary cracks and the formation of new cracks in the pillars between window openings. Unlike the walls without openings, the arrangement of cracks was not symmetrical. The reason was the presence of supports for window pillars and the value *σ*_c_. Cracks in the outer pillar at support B in the walls under minimum compression and shearing (*σ*_c_ = 0.1 N/mm^2^) propagated upwards (Figure 11a). In the model under maximum compression (*σ*_c_ = 1.0 N/mm^2^) and at increasing loading, cracks were observed in the central aera of the pillar and also on the side of the support B. The cracks were vertical in the extended head joints. A further increase in shear loading caused the development of symmetric cracks, which propagated towards the bottom and top edges of the wall (Figure 11b). Simultaneously, inclined cracks, which developed in the central area of the masonry and in the bottom (tension) corner of the window opening, were formed in the pillar on the side of support A. The failure process of unconfined models (*σ*_c_ = 0.1 N/mm^2^) was moderately gentle. Existing cracks increased their width and range. Damage at the time of failure covered the whole height of the pillars, and the width of cracks reached even 5 mm (Figure 11a). And the failure process in the walls subjected to maximum compression (*σ*_c_ = 1.0 N/mm^2^) was rather rapid. Apart from the development of the existing cracks in the central area of the pillar, masonry units in the support place for the lintel on the side of support B were crushed (Figure 11b).

**Figure 12:**
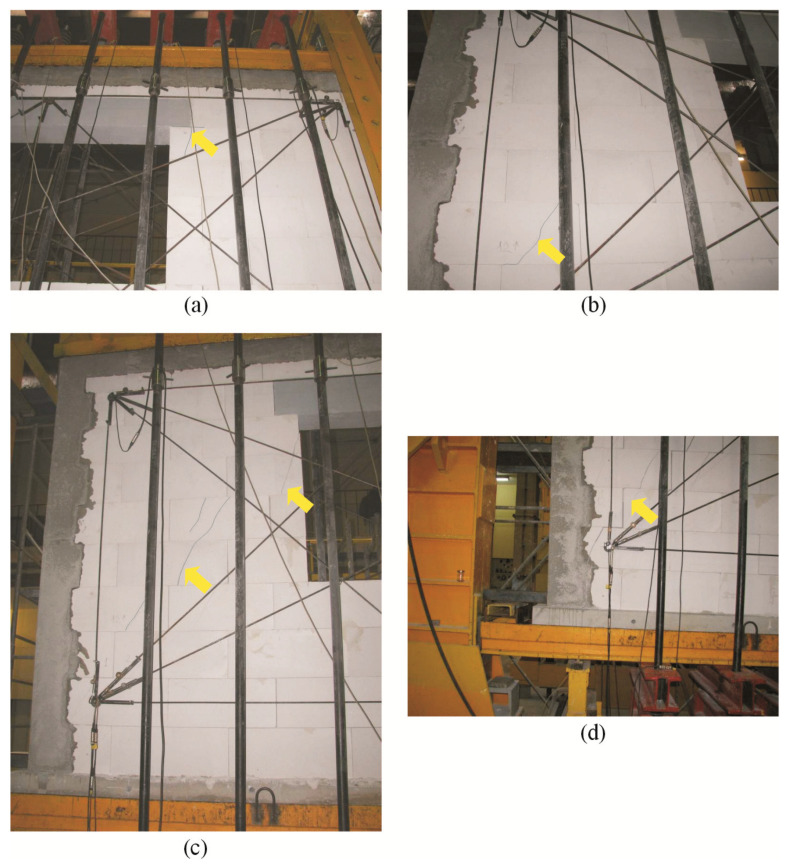
Superficial cracks in confined walls of the series HAS-C1-AAC under initial compressive stress up to *σ*_c_ = 0.1 N/mm^2^: (**a**) first cracks in the top corner of the pier; (**b**–**d**) secondary cracks in the bottom area of the pier at the side of support B.

In the confined walls of the series HAS-C1-AAC under minimum compression up to the value of 0.1 N/mm^2^, a first crack in the wall developed at the lintel support in the top area of the wall at the side of support A (Figure 12a), and then in the bottom area of the pier at the side of support B (Figure 12b). An increase in loading led to more superficial cracks in the bottom and central areas of the pier at the side of support B. Cracks were also formed at the lintel support (Figure 12c). The cracks were observed at almost the same time in the bottom corner of the wall above support B (Figure 12d).

**Figure 13:**
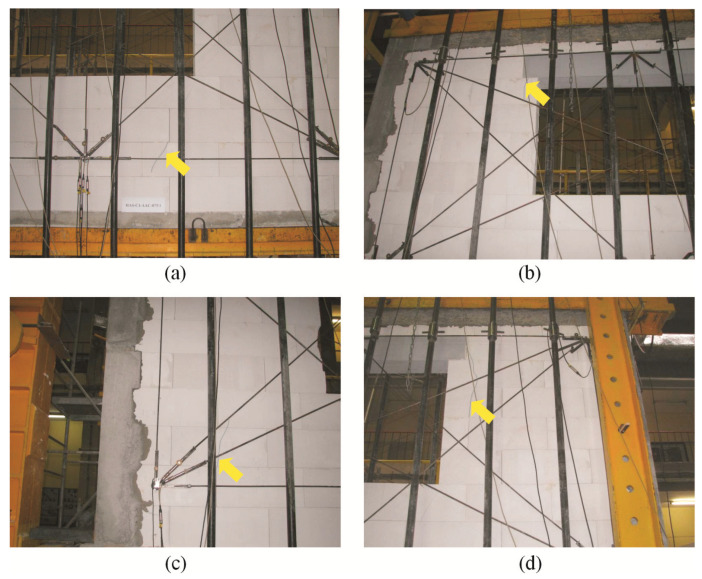
Superficial cracks in confined walls of the series HAS-C1-AAC under initial compressive stress up to *σ*_c_ = 0.75 N/mm^2^: (**a**) first cracks in the bottom corner of the pier; (**b**) first vertical cracks at the lintel support; (**c**) secondary cracks in the bottom area of the pier above support B; (**d**) vertical cracks at the lintel support.

**Figure 14:**
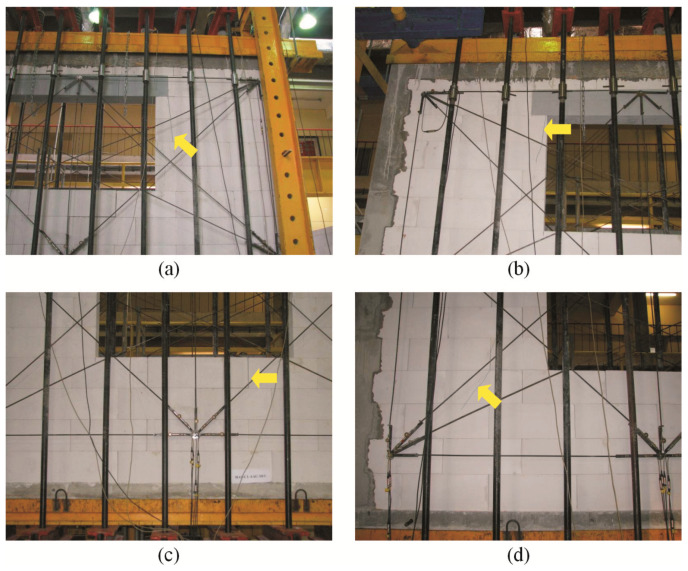
Superficial cracks in confined walls of the series HAS-C1-AAC under initial compressive stress up to *σ*_c_ = 1.0 N/mm^2^: (**a**) first cracks in the top corner of the pier; (**b**) first vertical cracks at the lintel support; (**c**) vertical cracks in the spandrel panel; (**d**) inclined cracks in the pier at the side of support B.

In the walls compressed to a value of 0.75 N/mm^2^, the first cracks developed in the top corner of the window opening at the side of support A (Figure 13a). Almost at the same time, vertical cracks were formed (near the lintel support) in the pier at the side of support B (Figure 13b). An increase in loading led to the formation of vertical and inclined cracks in the bottom part of the pier at the side of support B (Figure 13c). Similar cracks also developed in the pier at the side of support A (Figure 13d). In the walls compressed to the value of 1.0 N/mm^2^, a first crack developed along the vertical edge of the window opening at the side of support A (Figure 14a). Almost at the same time vertical cracks were formed at the lintel support on the side of support B (Figure 14b). An increase in loading led to the formation of vertical cracks in the spandrel panel (Figure 14c) or at the side of support B (Figure 14d).

**Figure 15:**
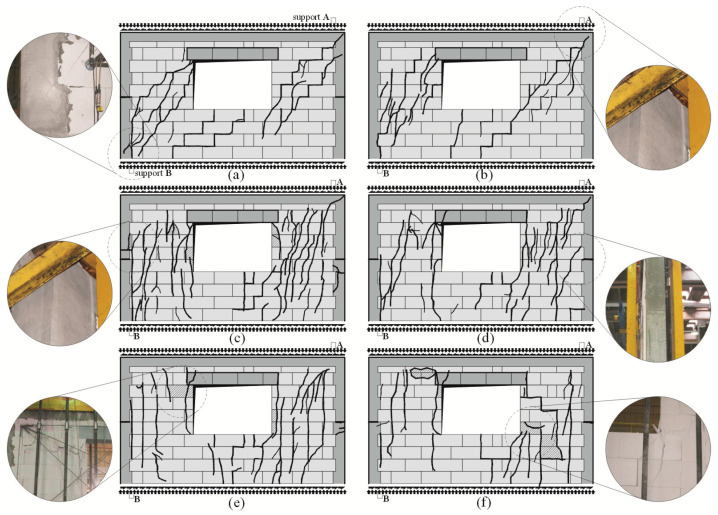
Images of cracks in confined walls of the series HAS-C1-AAC at failure: (**a**) the wall HAS-C1-AAC-010/1 under initial compressive stress up to *σ*_c_ = 0.1 N/mm^2^, (**b**) the wall HAS-C1-AAC-010/2 under initial compressive stress up to *σ*_c_ = 0.1 N/mm^2^, (**c**) the wall HAS-C1-AAC-075/1 under initial compressive stress up to *σ*_c_ = 0.75 N/mm^2^, (**d**) the wall HAS-C1-AAC-075/2 under initial compressive stress up to *σ*_c_ = 0.75 N/mm^2^, (**e**) the wall HAS-C1-AAC-10/1 under initial compressive stress up to *σ*_c_ = 1.0 N/mm^2^, (**f**) the wall HAS-C1-AAC-10/2 under initial compressive stress up to *σ*_c_ = 1.0 N/mm^2^.

In the models subjected to a minimum compressive load up to 0.1 N/mm^2^, inclined cracks developed and ran through the whole height of the opposite pier at the side of support A. Distinct cracks were observed in the bed and head joints in the spandrel panel. A reinforced core above support B was cracked in the connection with the bottom horizontal member (Figure 15a). A diagonal crack was found in the corner of the core at the side of support A (Figure 15b). In the walls under initial compressive stress up to 0.75 N/mm^2^ inclined cracks in the masonry were running through the whole height of the piers at the side of supports A and B. Horizontal cracks were found at the mid-height of the cores at the side of supports A and B (Figure 15c,d). Inclined cracks were also found in the spandrel panel. In the walls under maximum compressive stress up to the value of 1.0 N/mm^2^, cracks developed along vertical edges of the window opening at the side of support A, and additional cracks in the pier at the side of support B were running perpendicularly to vertical cores. At failure the top area of the masonry was crushed at the side of support B (Figure 15e). A similar situation was found in the bottom corner of the window opening at the side of the opening (Figure 15f).

**Figure 16:**
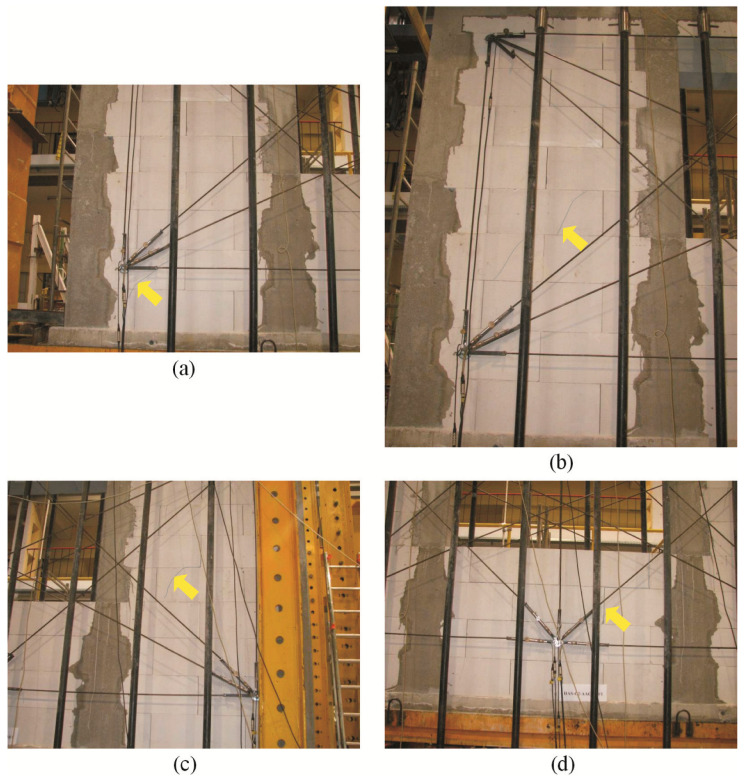
Superficial cracks in confined walls of the series HAS-C2-AAC under initial compressive stress up to *σ*_c_ = 0.1 N/mm^2^: (**a**) first cracks in the bottom area of the pier at the side of support B; (**b**) secondary cracks in the pier at the side of support B; (**c**) secondary cracks in the pier at the side of support A; (**d**) secondary cracks in the spandrel area.

In the confined walls of the series HAS-C1-AAC under minimum compression up to the value of 0.1 N/mm^2^, a first inclined crack in the wall developed in the bottom area of the pier at the side of the support B (Figure 16a). A slight increase in loading led to the formation of additional inclined cracks in the top area of the pier (Figure 16b). Cracks in the central part of the pier were at the side of support A (Figure 16c). Inclined cracks in the piers and the spandrel panel developed due to a further increase in loading (Figure 16d).

**Figure 17:**
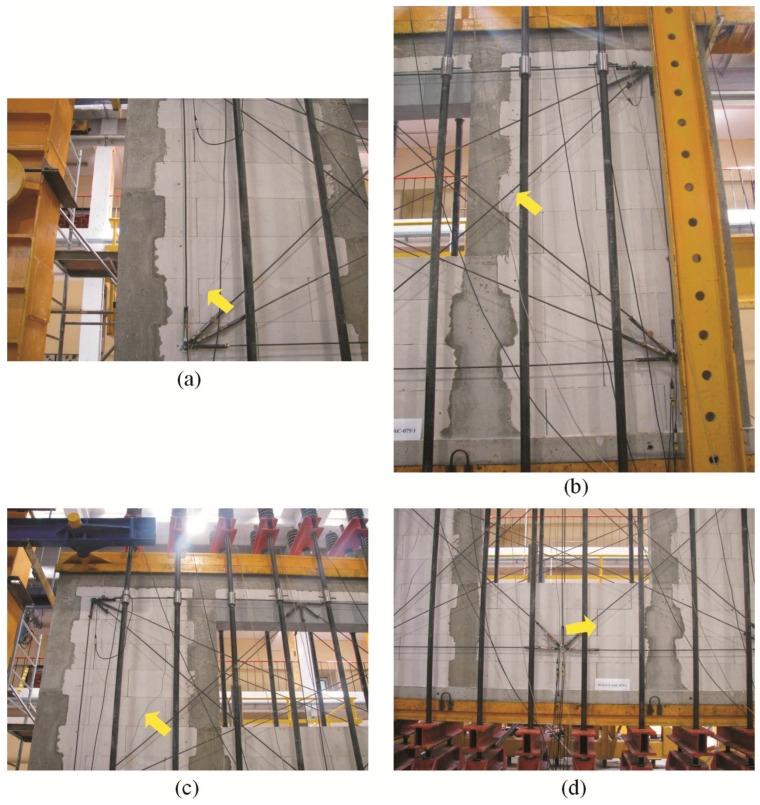
Superficial cracks in confined walls of the series HAS-C2-AAC under initial compressive stress up to *σ*_c_ = 0.75 N/mm^2^: (**a**) first cracks in the bottom corner of the pier; (**b**) first vertical cracks at the joint between confining elements and the masonry; (**c**) secondary cracks in the central area of the pier above support B; (**d**) vertical cracks in the spandrel panel.

In the walls compressed to a value of 0.75 N/mm^2^, a first inclined crack in the wall developed in the bottom area of the pier at the side of support B (Figure 17a). The first vertical cracks in the pier at the side of support B were at joint of the core surrounding the window opening (Figure 17b). With a slightly increasing load, inclined cracks formed at the pier bottom and ran towards the top internal corner of the window opening (Figure 17c). Additional cracks were mainly at joints between masonry units in the spandrel panel (Figure 17d).

**Figure 18:**
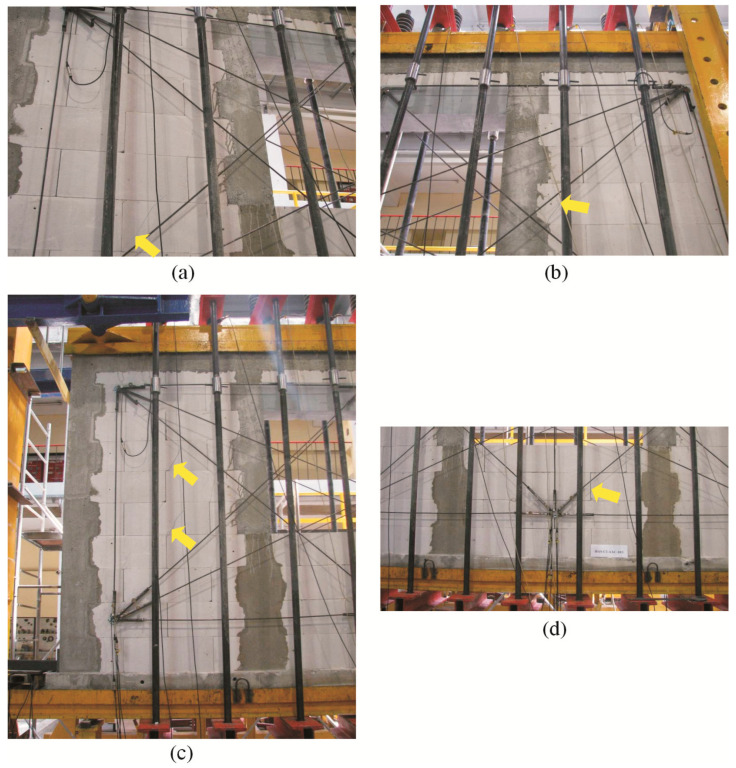
Superficial cracks in confined walls of the series HAS-C2-AAC under initial compressive stress up to *σ*_c_ = 1.0 N/mm^2^: (**a**) first cracks in the central part of the pier; (**b**) first vertical cracks at the joint between confining elements and the masonry; (**c**) secondary cracks in corners of confining elements in the pier above support B; (**d**) vertical cracks in head and bed joints in the spandrel area.

In the walls compressed to a value of 1.0 N/mm^2^, a first inclined crack in the wall developed in the central area of the pier at the side of support B (Figure 18a). The first cracks in the pier at the side of support A were at its mid-height, near the reinforced concrete core (Figure 18b). With a slightly increasing load, inclined cracks were formed in the pier at the side of support B, in the corners of the masonry units (Figure 18c). Furthermore, cracks were observed in the head and bed joints in the spandrel panel (Figure 18d).

**Figure 19:**
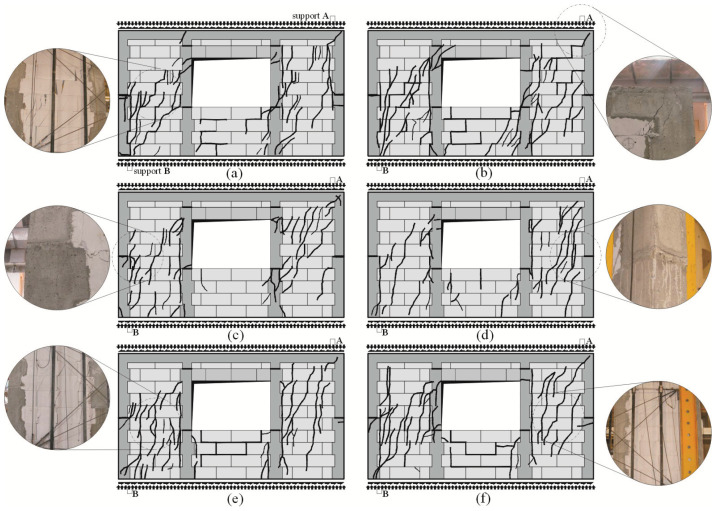
Images of cracks in confined walls of the series HAS-C2-AAC at failure: (**a**) the wall HAS-C2-AAC-010/1 under initial compressive stress up to *σ*_c_ = 0.1 N/mm^2^, (**b**) the wall HAS-C2-AAC-010/2 under initial compressive stress up to *σ*_c_ = 0.1 N/mm^2^, (**c**) the wall HAS-C2-AAC-075/1 under initial compressive stress up to *σ*_c_ = 0.75 N/mm^2^, (**d**) the wall HAS-C2-AAC-075/2 under initial compressive stress up to *σ*_c_ = 0.75 N/mm^2^, (**e**) the wall HAS-C2-AAC-10/1 under initial compressive stress up to *σ*_c_ = 1.0 N/mm^2^, (**f**) the wall HAS-C2-AAC-10/2 under initial compressive stress up to *σ*_c_ = 1.0 N/mm^2^.

As in the models with circumferential confinement, inclined cracks covering nearly the whole height of the pier at the side of support B were found in the models subjected to a minimum compression of 0.1 N/mm^2^ at failure. The intensity of cracking in the pier at the side of support B was slightly lower (Figure 19a). Horizontal cracks at the mid-height of the core at the sides of supports A and B and cracks in the corner were found (Figure 19b). Also, in the walls under initial compressive stress up to 0.75 N/mm^2^, inclined cracks in the masonry were running through the whole height of the piers at the sides of the supports A and B. However, their intensity was considerably higher than in the walls under minimum compression. Horizontal cracks were found at the mid-height of the cores at the sides of supports A and B (Figure 19c,d). Inclined and vertical cracks covered nearly the whole height of the piers at the sides of the support B (Figure 19e) and the support A (Figure 19f) in the walls under compression of 1.0 N/mm^2^ in the phase prior to failure. Furthermore, cracks developed along the joint between the vertical edges of the confining elements and the masonry. A horizontal crack at the joint between the core and the horizontal member developed in the bottom area of the edge core at the side of support B. A single, almost vertical crack was also formed in the top corner of the joint between the core and the horizontal member.

### 4.3. Stress-Strain Relationships

**Figure 20:**
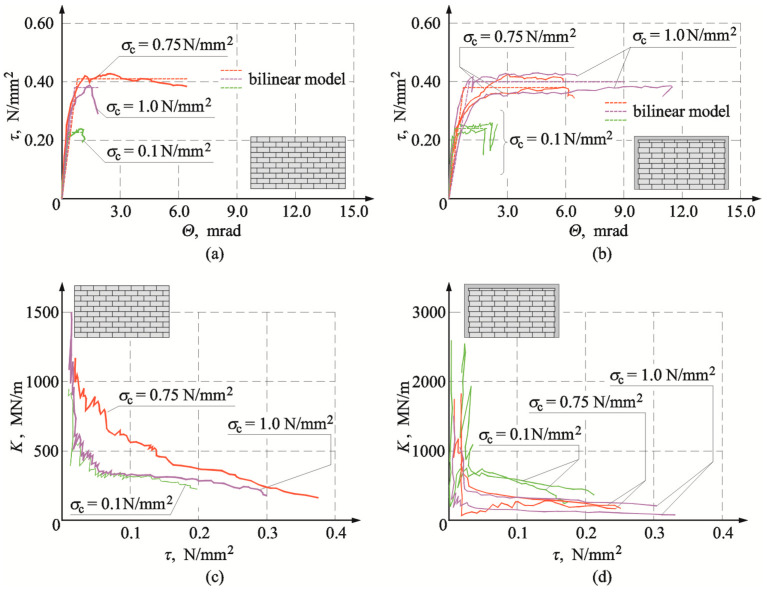
Comparison of experimental results for walls without opening acc. to [26]: (**a**) shear stress-strain angle relationship for walls of the series HOS-AAC; (**b**) shear stress-strain angle relationship for walls of the series HOS-C-AAC; (**c**) stiffness-shear stress relationship for walls of the series HOS-AAC; (**d**) stiffness-shear stress relationship for walls of the series HOS-C-AAC.

Figure 20a,b illustrates the determined stress τ_v,i_ and strain Θ*_i_* relationships for the reference models of the series HOS-AAC and for the models with confining elements of the series HOS-C-AAC (without openings). And Figure 20c,d shows changes in stiffness *K*_i_ in the function of shear stresses *τ*_v,i_ for the reference models without confinement and the models with circumferential confinement. For the reference models (without confinement) and the confined models, the determined relationships between shear stress and deformation were proportional until the time of cracking. Differences were observed in the phase of reaching maximum values of shear stress. For the reference model, under minimum compression of 0.1 N/mm^2^ and maximum compression up to 1.0 N/mm^2^, relatively sudden weakening was noticed after reaching maximum shear stresses. In the model subjected to initial compression of 0.75 N/mm^2^, plastic strains increased in that phase of loading. For the confined models, regardless of initial compressive stresses, shear stresses at the time of cracking corresponded to lower shear strains, and plastic strains increased after reaching the maximum values of shear stress. The greatest plastic strains were found for the model under maximum compression at a value of 1.0 N/mm^2^.

Changes in shear strains were also manifested in the determined relationships between stiffness and shear stress (Figure 20c,d). The tests on reference models demonstrated a significant degradation of the initial stiffness *K*_0_ in the initial phase of loading in the range of shear stresses from 0 to 0.05 N/mm^2^. An increase in shear stresses clearly reduced the stiffness. The confined models behaved in a very similar way. A clear difference was found in the initial phase of loading. The degradation of the initial stiffness *K*_0_ was more pronounced, and a further increase in shear stresses did not lead to such a clear reduction of stiffness as in the reference models.

The obtained results expressed as stresses *τ*_cr_ and *τ*_u_, shear strains *Θ*_cr,_ and shear deformations *Θ*_u_ are presented in Table 1. The table also includes the values of initial total stiffness *K*_0_ determined at shear stresses in the range of 0–0.05*τ*_u_ and at the time of wall cracking *K*_cr_.

**Figure 21:**
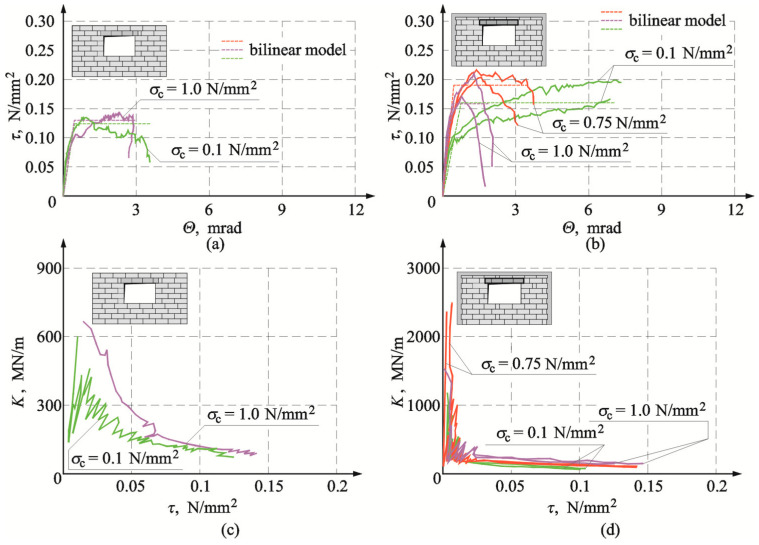
Comparison of experimental results for walls with openings: (**a**) shear stress-strain angle relationship for walls of the series HAS-AAC; (**b**) shear stress-strain angle relationship for walls of the series HAS-C1-AAC; (**c**) stiffness-shear stress relationship for walls of the series HAS-C1-AAC; (**d**) stiffness-shear stress relationship for walls of the series HAS-C1-AAC.

Figure 21a shows stress τ*_v,i_* strain *Θ_i_* relationships for elements of the series HAS-AAC with openings, whereas Figure 21b shows these relationships for confining elements of the series HAS-C1-AAC with openings. The obtained changes in total stiffness of all tested elements are presented in Figure 21c,d.

Relationships τ*_v,i_*–*Θ_i_* were almost proportional in all models until the moment of cracking. For the model marked as HAS-AAC-010 (Figure 21a), compressed to a value of 0.1 N/mm^2^, a slight curve depression was observed after cracking and then strengthening. The effect of strengthening was also noticed in the element HOS-AAC-10 subjected to maximum compression, but shear strain was similar to that in the model under minimum compression. In confined walls of the series HAS-C1-AAC initially compressed to 0.1 N/mm^2^ (Figure 21b), there was a significant drop in stiffness and a significant increase in non-dilatational strains, much greater than in the unconfined walls. No effect of weakening was observed when maximum compressive stress was reached. In the walls compressed to values of 0.75 N/mm^2^ and 1.0 N/mm^2^, the effect of strengthening was observed after cracking. However, weakening was found after reaching maximum values of stress, contrary to the models under minimum compressive stress.

**Figure 22:**
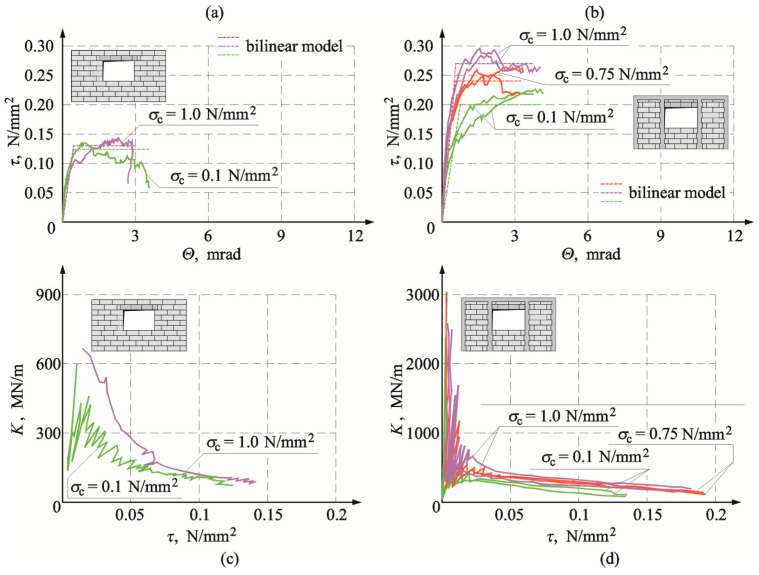
Comparison of experimental results for walls with openings: (**a**) shear stress-strain angle relationship for walls of the series HAS-AAC; (**b**) shear stress-strain angle relationship for walls of the series HAS-C2-AAC; (**c**) stiffness-shear stress relationship for walls of the series HAS-C2-AAC; (**d**) stiffness-shear stress relationship for walls of the series HAS-C2-AAC.

In the unconfined walls of the series HAS-AAC (Figure 21c), reduction of the stiffness *K_i_* was reversely proportional to an increase in shear stresses, and the greatest degradation of the initial stiffness *K*_0_ was found at shear stress τ*_v,i_* < 0.05 N/mm^2^. The trend in the confined walls was similar (Figure 21d). In the case of the unconfined walls, the reduction of stiffness within a range of shear stresses τ*_v,i_* > 0.05 N/mm^2^ was not as pronounced. Figure 22a illustrates the determined stress τ*_v,i_* strain *Θ_i_* relationships for elements of the series HAS-AAC with openings, whereas Figure 22b shows these relationships for confining elements of the series HAS-C2-AAC with openings. The obtained changes in total stiffness of all tested elements are presented in Figure 22c,d. Initial changes in *τ*_v,i_–*Θ*_i_ relationship did not considerably differ from the previously presented test results. In confined walls of the series HAS-C2-AAC initially compressed to 0.1 N/mm^2^ (Figure 22b), stiffness was reduced (an increase in the slope of curves), and that reduction was considerably lower than in the case of the models with perimeter confinement. Weakening was not observed after reaching maximum compressive stress. Strengthening of the models compressed to values of 0.75 N/mm^2^ and 1.0 N/mm^2^ was found after cracking. And after reaching maximum values of stress, the effect of weakening was not as definite as in the models with C-type confinement. The relationship between *K* and *τ* in confined walls (Figure 22d) was very similar to that in the case of the walls with C1-type confinement. There was also a very rapid drop in stiffness at the initial phase of loading and then a definite “flattening”.

The test results expressed as stresses at the time of cracking *τ*_cr_ and failure *τ*_u_, and the corresponding angles of strain *Θ*_cr_ and shear deformation *Θ*_u_, initial stiffness *K*_0_, and stiffness at the time of cracking *K*_cr_, are given in Table 2.

### 4.4. Effect of Wall Confinement

**Figure 23:**
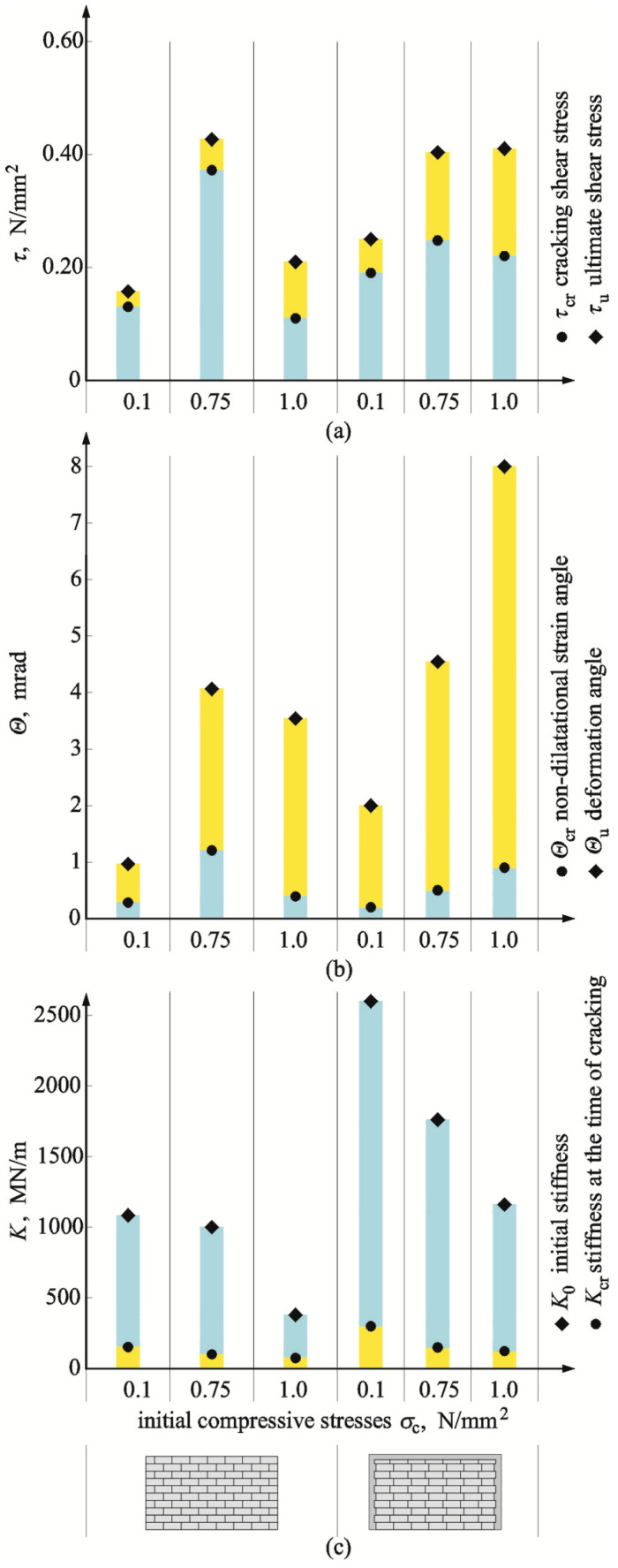
Comparison of test results for walls of the series HOS-C-AAC without openings and with and without confinement: (**a**) cracking and failure stress; (**b**) angles of shear strain and shear deformation; (**c**) initial stiffness and stiffens at the moment of cracking.

**Figure 24:**
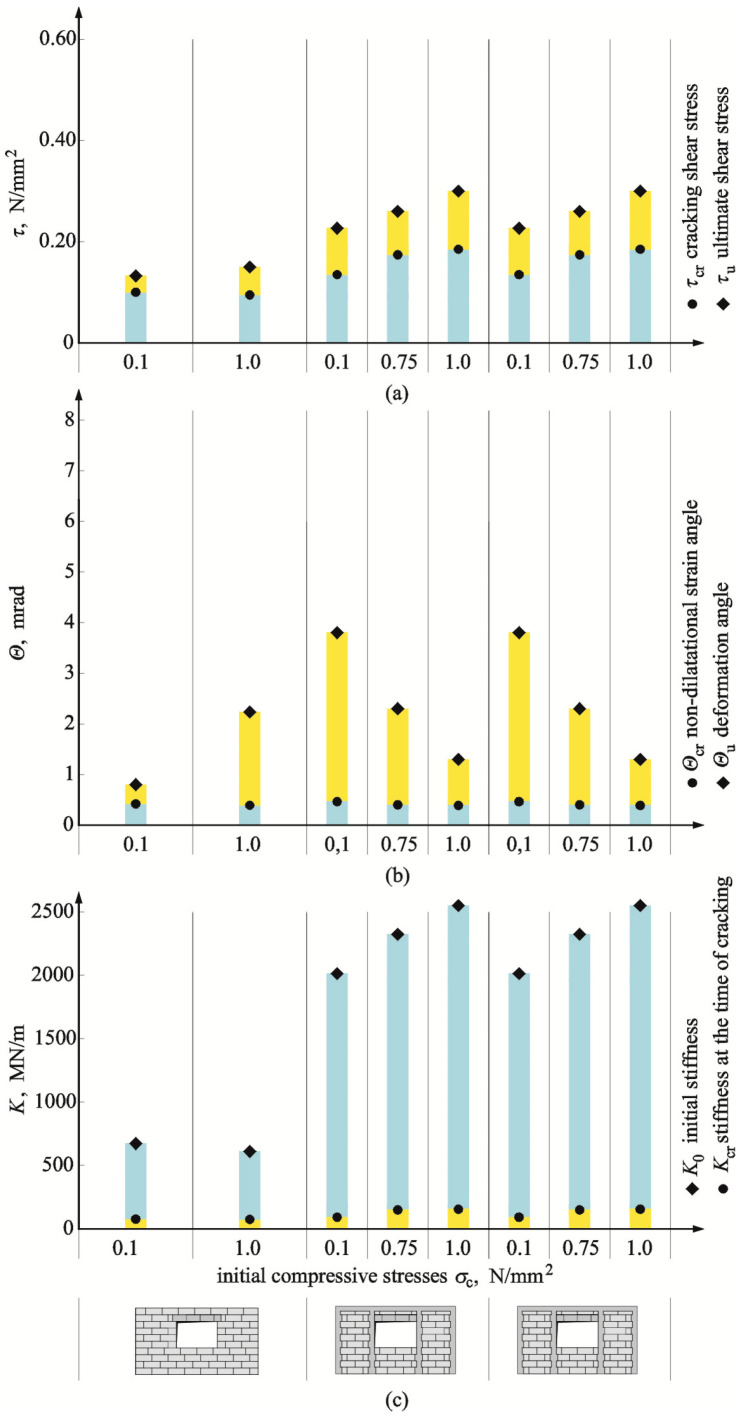
Comparison of test results for walls of the series HAS-C1-AAC and HAS-C2-AAC with openings and with and without confinement: (**a**) cracking and failure stress; (**b**) angles of shear strain and shear deformation; (**c**) initial stiffness and stiffening at the moment of cracking.

The mean results from testing the confined walls without openings (HOS-C-AAC) and with openings (HAS-C1-AAC i HAS-C2-AAC) were compared with the test results for the reference walls—the unconfined ones, which are presented in Table 3. Figure 23 and Figure 24 present the absolute vales of determined stresses, angles of shear deformation/strain, and initial stiffness of the reference (unconfined) and confined walls.

For the walls without openings (the series HOS-C-AAC) subjected to minimal values of initial compression [26], no increase in stress *τ*_cr_ was observed when compared to the stress values determined in the reference models. Stress *τ*_u_ increased only by 7% at the time of failure. In the walls under initial compression up to 0.75 N/mm^2^, the cracking stress determined for the confined walls was lower by 33% and by 5% at the time of failure than in the reference models. When shearing was accompanied by an initial compressive stress equal to 1.0 N/mm^2^, the stresses *τ*_cr_ and *τ*_u_ increased by 6%. Shear deformations *Θ*_cr_ corresponding to cracks visible in confined walls under minimum compression up to 0.75 N/mm^2^ were smaller by 25% and 32% when compared to deformations determined for the reference models. Deformations in the walls under maximum compression increased by 77%. Shear deformations *Θ*_u_ determined at the time of failure in the confined walls were each time greater than the ones determined for the unconfined models. Deformations were increasing with an increase in the value *σ*_c_ from ca. ~110% to 450%. In case of initial stiffness *K*_0_, an increase in initial compressive stress led to a pronounced reduction of stiffness. Stiffness was found to drop from 179% to 52% at an increase in compressive stresses from 0.1 to 0.75 N/mm^2^, and in the models under maximum compression, this value was lower by 23% than in the unconfined models. Stiffness *K*_cr_ in the confined models at the time of cracking was greater by 33% than in the walls under minimum compressive stress. An increase in initial compressive stresses *σ*_c_ from 0.75 N/mm^2^ to 1.0 N/mm^2^ reduced stiffness. In the model under maximum compression, stiffness was reduced by 23%.

For confined walls of the series HAS-C1-AAC with an opening and perimeter confinement, cracking stress observed under minimum initial compressive stress was slightly lower (7%) than in unreinforced walls. In walls compressed to a value of 1.0 N/mm^2^, cracking stress increased by more than 35% when compared to the unreinforced models. At failure of the walls compressed to the values of 0.1 and 1.0 N/mm^2^, an increase in ultimate stress was 33% and 36% when compared to the models without reinforcement. At the time of cracking in confined walls compressed to 0.1 N/mm^2^, values of shear strain angle were greater by ca. 17% when compared to the unconfined walls. However, where shearing was accompanied by the maximum compressive stress, shear strain was reduced by more than 24%. The greatest variation in test results was observed at failure. Shear strains were more than 9-fold greater in the models under minimum compression than in the reference models. For the models compressed to a value of 1.0 N/mm^2^, shear strain dropped by more than 53%. Initial stiffness of walls was greater than in unconfined walls under compressive stresses of 0.1 N/mm^2^ and 1.0 N/mm^2^ by 65% and 83%, respectively. The stiffness *K*_cr_ of the models subjected to minimum compressive stress at the time of cracking was lower by ca. 20% than that of the reference models, and an increase in stiffness exceeded 83% in the models under maximum compressive stress.

For confined walls of the series HAS-C2-AAC with an opening and confinement along the perimeter and vertical edges of the openings, cracking stress observed under minimum initial compressive stress was greater by 22% than in the unreinforced walls. In walls compressed to a value of 1.0 N/mm^2^, cracking stress increased by more than 89% when compared to the unreinforced models. At failure of the walls compressed to the values of 0.1 and 1.0 N/mm^2^, an increase in ultimate stress was 68% and 105% when compared to the models without reinforcement. At the time of cracking in confined walls compressed to 0.1 N/mm^2^, values of shear strain angle were greater by ca. 12% when compared to the unconfined walls. However, where shearing was accompanied by the maximum compressive stress, shear strain was slightly reduced by 7%. The greatest variation in test results was observed at failure. Shear strains were nearly 5-fold greater in the models under minimum compression than in the reference models. For the models compressed to a value of 1.0 N/mm^2^, shear strain dropped by more than 20%. Initial stiffness of walls was greater than in unconfined walls under compressive stresses of 0.1 N/mm^2^ and 1.0 N/mm^2^ by 204% and 324%, respectively. Stiffness *K*_cr_ in all the models at the time of cracking was greater than in the reference models—by 11% in the model under minimum compressive stress and by 112% in the walls subjected to maximum compressive stress.

## 5. Analysis of Test Results

The morphology of cracks and the failure process of unconfined walls without openings did not differ significantly from those presented in the papers [1,2] and the direction was consistent with the direction of the main tensile stresses. Cracks in the walls with openings were also initiated in the tension corners of window openings [3,4,5] and then covered piers between openings. A significant difference was observed in confined walls, in which vertical cores ran along the vertical edges of openings. The first cracks did not develop in the tension corners of openings but in the corners of piers. A significantly greater increase in shear strain at failure caused more dramatic cracking in the masonry and confining reinforced concrete elements.

**Figure 25:**
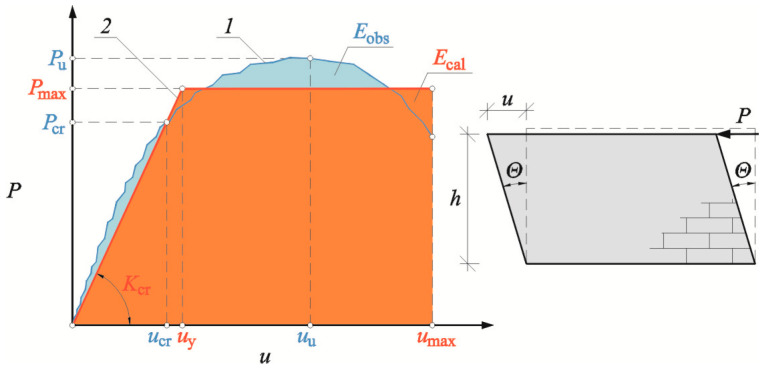
Symbols for wall behavior used in the bilinear model; *1*—test results, *2*—bilinear idealization.

The relationships between load and displacement of the analyzed confined walls were characterized by strong non-linearity, which could be difficult for interpretation and practical application. Therefore, confined masonry walls with and without openings were described with a bilinear (two-linear) relationship between load and displacement [47]. The proposed model (Figure 25) had an elastic branch connecting the beginning of the coordinate system with the point corresponding to plastic displacements *u*_y_ and maximum force *P*_max_. The straight line is going through the point with coordinates (*u*_cr_; *P*_cr_) corresponding to a crack. A horizontal branch of the model corresponds to the force *P*_max_ and is adequate for the range of displacements corresponding to softening *u*_y_ to maximum displacements *u*_max_. The following parameters are required to describe the model: *K*_cr_—stiffness at the time of cracking; *u*_max_—maximum displacement at failure of the wall model; and *P*_max_—maximum force. Two first parameters were determined from tests on walls, and the force *P*_max_ was determined similarly as in the papers [48,49] on the basis of the dissipated energy of the wall *E*_obs_ calculated from the following Equation (5). Assuming that the dissipated energy determined during the tests, *E*_obs_ is equal to the energy of the bilinear model, *E*_cal_, the following relationship can be developed:

(6) $$E_{\mathrm{obs}}=E_{\mathrm{cal}},$$

which gives the following equation:

(7) $$E_{\mathrm{obs}}=E_{\mathrm{cal}}=\frac{1}{2}P_{\max}\left[u_{\max}+\left(u_{\max}-u_{y}\right)\right].$$

Assuming that:

(8) $$u_{y}=\frac{P_{\max}}{K_{\mathrm{cr}}},$$

The following relationship is obtained:

(9) $$\begin{array}{l} E_{\mathrm{obs}}=\frac{1}{2}P_{\max}\left[u_{\max}+\left(u_{\max}-u_{y}\right)\right]=\frac{1}{2}P_{\max}\left(2u_{\max}-u_{y}\right)=\frac{1}{2}P_{\max}\left(2u_{\max}-\frac{P_{\max}}{K_{\mathrm{cr}}}\right)\to \\ P_{\max}^{2}-2u_{\max}K_{\mathrm{cr}}P_{\max}+2E_{\mathrm{obs}}K_{\mathrm{cr}}=0. \end{array}$$

The acceptable root of the quadratic equation specifies the maximum force equal to:

(10) $$P_{\max}=u_{\max}K_{\mathrm{cr}}-\sqrt{{\left(u_{\max}K_{\mathrm{cr}}\right)}^{2}-2E_{\mathrm{obs}}K_{\mathrm{cr}}}.$$

where *u*_max_ = *Θ*_max_*h*, *Θ*_max_—maximum angle of shear deformation corresponds to the failure of the wall.

The coefficient describing the yield strength of the wall is expressed by the ratio of maximum displacement *u*_max_ and displacement corresponding to softening *u*_y_, calculated from the following formula:

(11) $$\mu =\frac{u_{\max}}{u_{y}}\geq 1.0$$

where *u*_y_ = *P*_max_/*K*_cr_.

The coefficient *μ* = 1 describes the (elastic-brittle) material without a yield plateau, and the material is ductile when *μ* > 1. The coefficient is *μ* →∞ for perfectly elastic and plastic materials.

Determined parameters for bilinear models of confined walls are compared in Table 4, and Table 5 presents test results for reference walls without confinement. Table 6 shows results compared with the results for reference models. Figure 20, Figure 21 and Figure 22 present a comparison of test results for the relationship between shear stress and shear strain.

Dissipated energy in walls without openings was increasing nearly proportionally to displacements *u*_y_ and *u*_max_ with increasing values of initial compressive stress. The corresponding coefficient of ductility varied within a range from 7.28 to 8.18, which means that this type of wall was characterized by substantial plastic behavior. When compared to reference unconfined models, the determined values of dissipated energy were greater on average by 200%, and the values of the coefficient of ductility were the greatest in the models under maximum compression (greater on average by 109%). The confined walls with openings presented a considerable drop in dissipated energy in comparison to the models without openings. When compared to the unconfined models, the mean maximum forces *P*_max_ determined for the confined walls did not increase (values ranged from 0.93 to 1.06).

Energy in the models of the series HAS-C1-AAC with circumferential confinement was decreasing with increasing values of initial compressive stress, and the coefficient of ductility dropped from 9.45 to 5.09. The mean energy increased by 68% when compared to unconfined models, and the biggest increase was noticed for the models under minimum compression (2.57). Similarly, the mean determined ratio of coefficients of ductility was greater by ca. 13%. In that case, the maximum force *P*_max_ increased on average by 23% (from 1.20 to 1.26).

Confinement along the vertical edges of the window opening in the models of the series HAS-C2-AAC did not cause a substantial increase in dissipated energy nor the coefficient of ductility, which varied from 5.48 to 6.40. The effect of confinement was observed for unconfined masonry. An increase in mean dissipated energy was 126%, while the coefficient of ductility was not considerably changed. Following the same procedure as for the previous models, the ratio of maximum forces *P*_max_ was determined for the model with and without confinement, which showed an increase of an order of 77% (from 1.65 to 1.89). This case confirmed that the recommendation specified in the standard EN-1996-1-1 [37], according to which openings having an area of 1.5 m^2^ or more should be confined, was appropriate and desired.

The plastic behavior of confined structures increases the amount of dissipated energy, and therefore vibration damping is greater and beneficially reduces the values of inertia forces. The ductility coefficient for unconfined models was greater on average by 100%. However, absolute values of plastic displacement were increasing with increasing values of compressive stress in walls both with and without openings. Similar results were presented in the paper [50], in which the ductility of squat walls increased while stiffness degradation was reduced. The tests on slender walls [51] demonstrated that plastic strains decreased with increasing values of initial compressive stress. The walls of the series HAS-C2-AAC, which were confined along the vertical edges of openings, demonstrated a desirable increase in plastic strains, as in the case of the tests [33], by 26% more than in the unreinforced walls.

## 6. Conclusions

The following conclusions can be drawn on the basis of tests performed on confined walls:

- The observed processes of destruction of shear masonry with confinement indicate that:
  ○ Cracks in the models of the series HAS-C1-AAC with openings were formed in the tension corners of openings and then in the corners of window piers. At failure, inclined cracks in the piers and corners of the wall and confining elements were found in construction joints;
  The morphology of cracks in the models of the series HAS-C2-AAC was significantly different because the first cracks were formed in the bottom corners of the window piers (no signs of cracks in tension corners of the window openings), and an increase in loads led to crack formation at the interface with confinement and in spandrel areas.

- Regarding the shear stresses at the time of cracking *τ*_cr_ and failure *τ*_u_, the following observations were made:
  ○ In the models of the series HAS-C1-AAC with an opening and circumferential confinement, subjected to maximum compression, cracking stress at failure increased by nearly 35% when compared to the unconfined models. The maximum stress of confined models was greater in each case by 36% and 33%;
  ○ The applied confinement along the vertical edges of the models of the series HAS-C2-AAC led to an increase in cracking stress from 22% to 89%, regardless of values of initial compressive stress. A similar trend was found for maximum stresses, which increased within a range of 68–105%.

- Regarding shear strain angles at the time of cracking *Θ*_cr_ and failure *Θ*_u_, the following observations were made:
  ○ In the models of the series HAS-C1-AAC (circumferential confinement), deformations at the moment of cracking in the model subjected to minimum compression were greater by 17% than in the unconfined model. The angles of shear deformation in the models under maximum compression were narrower than in the unconfined models;
  ○ A similar trend was found near openings in the confined models of the series HAS-C2-AAC. Only in the model under minimum compressive stress did shear deformation at the time of cracking increase by ca. 12% and by 388% when subjected to maximum stress. Even under the greatest initial compressive stresses, deformations were smaller than in the unconfined models analysed in a similar way.

- Considering the initial stiffness *K*_0_ and stiffness at the time of cracking *K*_cr_, it was found that:
  ○ Only in the model under minimum compressive stress did stiffness at the moment of cracking increase by ca. 33%. In other models, values of stiffness did not dramatically differ or demonstrate lower stiffness than in the unconfined wall;
  ○ Initial compressive stress in the models of the series HAS-C1-AAC increased by 65–83%. That tendency was a bit different at the time of the cracking. An increase in stiffness was 83% only in the model under maximum compression, and in other models stiffness was lower than in the unconfined models;
  ○ In the elements of the series HAS-C2-AAC, initial stiffness tended to increase when compared to the unconfined models; however, an increase in stiffness was between 204% and 304%. At the moment of cracking, stiffness determined in the same way increased in every case by 11% and 112%.

The proposed bilinear model of the behavior of shear walls was based on the equivalence of dissipated energy determined from the tests and calculated for the model. Experimentally determined stiffness *K*_cr_ (at the moment of cracking) and maximum displacement of the wall determined at failure *u*_max_ were used to determine maximum force *P*_max_, plastic displacement *u*_y_ and the coefficient of ductility μ, which were compared with parameters for unconfined walls determined in the same way. The above aspects led to the following conclusions:

- Considering dissipated energy *E*_obs_, it was found that:
  ○ An increase in initial values of compressive stress in the unconfined models of the series HOS-C-AAC caused a clear increase in values of dissipated energy. The energy increased by more than 200% when compared to the unconfined models;
  ○ A situation in the elements of the series HAS-C1-AAC with circumferential confinement was the same as in the models without openings, and a mean increase in dissipated energy was above 68% when compared to the elements without confinement;
  ○ Confinement along openings in the models of the series HAS-C2-AAC followed an already observed trend, and the energy increased by 77% when compared to elements without confinement.

- Considering maximum force *P*_max_ it was found that:
  ○ In the walls of the series HOS-C-AAC an increase in initial compressive stresses did not cause an increase in maximum force when compared to the unconfined models;
  ○ A similar trend was noticed in the models of the series HAS-C1-AAC with confinement along circumference, and a mean increase of maximum force was 23%,
  ○ No significant changes were observed in the models of the series HAS-C2-AAC, and a mean increase of shear force *P*_max_ was 77%.

- Considering the coefficient of ductility *μ* it was found that:
  ○ In the walls of the series HOS-C-AAC an increase in initial compressive stresses had a significant effect on the coefficient of ductility, and a mean increase in ductility was 109% when compared to the unconfined models;
  ○ In the walls of the series HAS-C1-AAC with C1-type confinement, an increase in initial compressive stress led to the reduced coefficient of ductility, and the trend similar to the reference walls was observed. Ductility of the confined walls was greater by 13% when compared to the unconfined walls;
  ○ An increase in initial compressive stress in test elements of the series HAS-C2-AAC also reduced the coefficient of ductility. In that case ductility of confined walls was lower by 7% when compared to the reference models.

- Considering recommendations specified in the standard EN-1996-1-1 [37], according to which circumferential confinement is required for all openings with an area greater than 1.5 m^2^, for the walls without confinement it was found that:
  ○ Confining reinforced concrete elements along vertical edges of openings eliminated the formation of cracks in tensions corners of openings, which led to a clear increase in wall stiffness;
  ○ Confinement increased plastic displacements *u*_y_ by 17% on average, and maximum displacements *u*_max_ by 18%;
  ○ Maximum force *P*_max_ corresponding to softening increased by more than 45%;
  ○ Ductility of the models with confinement recommended by the standard EN-1996-1-1 dropped slightly by ca. 8%;
  ○ No confinement in the spandrel area could result in too early cracking in that part of the wall.

This paper is a continuation of the research performed by the author [26,45,46] and presents the experimental part of the research conducted at the Silesian University of Technology. Other analyses include numerical and analytical models of the behavior of confined walls to provide a safe prediction of parameters that determine the safety of a structure. The authors are aware that the number of analyzed models cannot provide quantitative conclusions, and are used to draw only qualitative conclusions. Further experimental work will focus on models with complete confinement around a window opening.

## Figures and Tables

**Table 1:** Test results for masonry walls without openings acc. to [26].

Series	Description	*σ*_c_ N/mm^2^	Stresses		Angles of Non-Dilatational Strain (Deformation)		Total Stiffness	
			Cracking	Failure	Cracking	Failure	Initial	At the Time of Cracking
			*τ*_cr_ N/mm^2^	*τ*_u_ N/mm^2^	*Θ*_cr_ mrad	*Θ*_u_ mrad	*K*_0_ MN/m	*K*_cr_ MN/m
HOS- AAC	Unconfined walls without reinforcement	0.1	0.196	0.235	0.281	0.97	932	229
		0.75	0.372	0.426	0.724	2.44	1168	169
		1.0	0.298	0.385	0.524	1.45	1541	187
HOS-C-AAC	Confined walls	0.1	0.213	0.260	0.191	2.234	2588	366
		0.1	0.168	0.242	0.229	1.813	2606	242
		0.75	0.252	0.425	0.499	3.039	1741	166
		0.75	0.245	0.386	0.482	5.879	1805	167
		1.0	0.331	0.387	1.380	11.494	871	79
		1.0	0.303	0.431	0.472	4.505	1506	210

**Table 2:** Test results for masonry walls with an opening.

Series	Description	*σ*_c_ N/mm^2^	Stresses		Angles of Non-Dilatational Strain (Deformation)		Total Stiffness	
			Cracking	Failure	Cracking	Failure	Initial	At the Time of Cracking
			*τ*_cr_ N/mm^2^	*τ*_u_ N/mm^2^	*Θ*_cr_ mrad	*Θ*_u_ mrad	*K*_0_ MN/m	*K*_cr_ MN/m
HAS- AAC	Unconfined walls without reinforcement	0.1	0.11	0.136	0.424	0.774	669	84.9
		1.0	0.097	0.144	0.422	2.237	602	75.6
HAS-C1-AAC	Confined walls C1-type confinement	0.1	0.101	0.168	0.486	6.900	1192	68.1
		0.1	0.104	0.202	0.507	7.327	1017	67.5
		0.75	0.133	0.218	0.376	1.378	2372	116
		0.75	0.140	0.205	0.443	1.578	2507	104
		1.0	0.138	0.211	0.332	1.323	657	136
		1.0	0.124	0.172	0.291	0.769	1540	140
HAS-C2-AAC	Confined walls C2-type confinement	0.1	0.135	0.225	0.413	3.745	2329	107
		0.1	0.133	0.229	0.538	3.812	1746	80.9
		0.75	0.191	0.253	0.535	2.045	3036	117
		0.75	0.158	0.265	0.295	2.572	1635	176
		1.0	0.182	0.297	0.316	1.505	2593	189
		1.0	0.186	0.294	0.466	2.080	2506	131

**Table 3:** Compared test results for confined and unconfined (reference) walls.

Series	Description	*σ*_c_ N/mm^2^	Stresses		Angles of Non-Dilatational Strain (Deformation)		Total Stiffness	
			Cracking	Failure	Cracking	Failure	Initial	At the Time of Cracking
			$\frac{\tau_{\mathrm{cr},C}}{\tau_{\mathrm{cr},U}}$	$\frac{\tau_{u,C}}{\tau_{u,U}}$	$\frac{\Theta_{\mathrm{cr},C}}{\Theta_{\mathrm{cr},U}}$	$\frac{\Theta_{u,C}}{\Theta_{u,U}}$	$\frac{K_{0,C}}{K_{0,U}}$	$\frac{K_{\mathrm{cr},C}}{K_{\mathrm{cr},U}}$
HOS-C- AAC	Confined Walls [26]	0.1	0.97	1.07	0.75	2.09	2.79	1.33
		0.75	0.67	0.95	0.68	1.83	1.52	0.99
		1.0	1.06	1.06	1.77	5.50	0.77	0.77
HAS-C1- AAC	Confined walls C1-type confinement	0.1	0.93	1.36	1.17	9.20	1.65	0.80
		1.0	1.35	1.33	0.74	0.47	1.83	1.83
HAS-C2- AAC	Confined walls C2-type confinement	0.1	1.22	1.68	1.12	4.88	3.04	1.11
		1.0	1.89	2.05	0.93	0.80	4.24	2.12

*τ*_cr,C_, *τ*_u,C_, *Θ*_cr,C_, *Θ*_u,C_, *K*_0,C_, *K*_cr,C_—test results for confined walls, *τ*_cr,U_, *τ*_u,U_, *Θ*_cr,U_, *Θ*_u,U_, *K*_0,U_, *K*_cr,U_—test results for unconfined (reference) walls.

**Table 4:** Parameters of the bilinear model of the confined walls.

Series	Description	*σ*_c_ N/mm^2^	Maximum Angle of Shear Deformation *Θ*_max_ mrad	Maximum Horizontal Displacement *u*_max_, mm	Dissipated Energy *E*_obs_ kJ	Maximum Force *P*_max_, kN	Horizontal Displacement *u*_y_, mm	$\mathrm{Ductility}\;\;\mathrm{Coefficient}\;\mu =\frac{u_{\max}}{u_{y}}$
HOS-C- AAC	Confined Walls [26]	0.1	1.95	4.74	0.846	191	0.651	7.28
		0.75	5.99	14.5	4.10	301	1.81	8.01
		1.0	9.05	22.0	6.42	316	2.69	8.18
HAS-C1-AAC	Confined walls C1-type confinement	0.1	7.12	17.3	2.04	124	1.83	9.45
		0.75	3.44	8.35	1.16	150	1.36	6.14
		1.0	1.89	4.58	0.518	124	0.90	5.09
HAS-C2-AAC	Confined walls C2-type confinement	0.1	3.97	9.64	1.43	163	1.76	5.48
		0.75	3.26	7.91	1.41	195	1.38	5.73
		1.0	3.67	8.90	1.77	216	1.39	6.40

**Table 5:** Parameters of the bilinear model of the unconfined walls.

Series	Description	*σ*_c_ N/mm^2^	Maximum Angle of Shear Deformation *Θ*_max_ mrad	Maximum Horizontal Displacement *u*_max_, mm	Dissipated Energy *E*_obs_ kJ	Maximum Force *P*_max_, kN	Horizontal Displacement *u*_y_, mm	$\mathrm{Ductility}\;\mathrm{Coefficient}\;\mu =\frac{u_{\max}}{u_{y}}$
HOS- AAC	Unconfined Walls [26]	0.1	1.04	2.52	0.393	186	0.812	3.10
		0.75	6.79	16.5	5.04	325	1.93	8.55
		1.0	1.80	4.36	1.06	299	1.60	2.73
HAS- AAC	Confined walls unconfined	0.1	3.55	8.61	0.794	99	1.16	7.42
		1.0	2.89	7.01	0.652	103	1.36	5.15

**Table 6:** Comparison of test results for the bilinear models of unconfined and confined walls.

Series	Description	*σ*_c_ N/mm^2^	Maximum Angle of Shear Deformation $\frac{\Theta_{\max,C}}{\Theta_{\max,U}}$	Maximum Horizontal Displacement $\frac{u_{\max,C}}{u_{\max,U}}$	Dissipated Energy $\frac{E_{\mathrm{obs},C}}{E_{\mathrm{obs},U}}$	Maximum Force $\frac{P_{\max,C}}{P_{\max,U}}$	Horizontal Displacement $\frac{u_{y,C}}{u_{y,U}}$	Ductility Coefficient $\frac{\mu_{C}}{\mu_{U}}$
HOS-C- AAC	Confined Walls [26]	0.1	1.88	1.88	2.15	1.03	0.80	2.34
		0.75	0.88	0.88	0.81	0.93	0.94	0.94
		1.0	5.03	5.03	6.03	1.06	1.68	2.99
Mean:			2.60	2.60	3.00	1.00	1.14	2.09
HAS-C1- AAC	Confined walls C1-type confinement	0.1	2.01	2.01	2.57	1.26	1.57	1.28
		1.0	0.65	0.65	0.79	1.20	0.66	0.99
Mean:			1.33	1.33	1.68	1.23	1.12	1.13
HAS-C2- AAC	Confined walls C2-type confinement	0.1	1.12	1.12	1.80	1.65	1.51	0.74
		1.0	1.13	1.13	2.71	1.89	1.01	1.11
Mean:			1.12	1.12	2.26	1.77	1.26	0.93

*Θ*_max,C_, *u*_max,C_, *E*_obs,C_, *P*_max,C_, *u*_y,C_, *μ*_y,C_—test results for confined walls, *Θ*_max,U_, *u*_max,U_, *E*_obs,U_, *P*_max,U_, *u*_y,U_, *μ*_y,U_—test results for unconfined (reference) walls.

## Data Availability

The raw data cannot be shared at this time as the data also form part of an ongoing study.
